# Treatment outcome and prognostic factors in PCNSL

**DOI:** 10.1186/s13000-019-0833-1

**Published:** 2019-06-13

**Authors:** Pimjai Niparuck, Paisarn Boonsakan, Taksayut Sutthippingkiat, Sulada Pukiat, Pichika Chantrathammachart, Sithakom Phusanti, Kochawan Boonyawat, Teeraya Puavilai, Pantep Angchaisuksiri, Artit Ungkanont, Suporn Chuncharunee, Vichai Atichartakarn

**Affiliations:** 1 Division of Hematology, Department of Medicine, Ramathibodi Hospital, Mahidol University, Bangkok, Thailand; 2 Department of Pathology, Ramathibodi Hospital, Mahidol University, Bangkok, Thailand; 3 Department of Medicine, ChakriNaruebodindra Medical Institute, Mahidol University, Bangkok, Thailand

**Keywords:** PCNSL, Chemotherapy plus WBRT, GCB lymphoma, Non GCB lymphomas, CD10

## Abstract

**Objectives:**

Standard treatment with a thiotepa-based regimen in countries with a limited resource is less feasible. Aims of the study were to evaluate the treatment outcome, and identify the prognostic factors in patients with primary central nervous system lymphoma (PCNSL).

**Methods:**

We conducted a retrospective study of 43 patients diagnosed with PCNSL, DLBCL subtype, who were treated with either HDMTX-based regimen, whole brain radiotherapy (WBRT), or both between 2010 and 2017.

**Results:**

There were 43 patients with a median age of 65 years (range 34–89 years). Protein expression of CD10, Bcl6, MUM1, Bcl2 and MYC were found in 19, 86, 91, 91 and 23%, respectively. Both germinal center B cell (GCB) and double-expressor (MYC+/Bcl2+) lymphomas were found in 21%. Multiple brain lesions and maximum tumor diameter (MTD) ≥5 cm were seen in 27 and 10 patients, respectively. Chemotherapy combined with WBRT, chemotherapy and WBRT were given to 20, 14 and 9 patients, respectively. Overall complete remission (CR) rate was 55.8%. Those receiving a combined-modality therapy had a higher CR rate than those treated with either chemotherapy (75% versus 36%, *p =* 0.036) or WBRT (75% versus 44%, *p =* 0.109). Median follow-up time was 17 months, and a 7-year overall survival (OS) was 40%. Features associated with a prolonged OS were an ECOG score ≤ 2 (*p* = 0.001), multiple brain lesions (*p* = 0.010), multiple area of brain involvement (*p* = 0.023), MTD < 5 cm (*p* = 0.004), GCB subtype (*p* = 0.003) and positive CD10 staining (*p* = 0.007). Expression of Bcl2 protein was associated with a significantly worse OS in the non-GCB DLBCL patients.

**Discussion:**

The factors affecting treatment outcomes in PCNSL were cell of origin of DLBCL, lesion characteristics, patients’ status and treatment regimen.

## Introduction

Primary central nervous system lymphoma (PCNSL) is an uncommon type of lymphoma, and has a dismal survival rate. It constitutes approximately 2–3% of non-Hodgkin’s lymphoma (NHL) cases, and non-Germinal-Center B cell (non-GCB) subtype of diffuse large B cell lymphoma (DLBCL) is the most common histopathology [1–4]. Prognostic features as proposed by the International Extra-nodal Lymphoma Study Group (IELSG) include age, Eastern Co-operative Oncology Group (ECOG) performance status score, serum lactate dehydrogenase (LDH) level, cerebrospinal fluid (CSF) protein concentration and involvement of deep structure of the brain [5, 6]. Treatment with chemotherapy and whole brain radiotherapy (WBRT) has a better survival rate than those treated with either modality alone. However, neurological toxicity was found in those receiving a combined-modality therapy after a long term follow-up [7–11]. The current chemotherapy for PCNSL is a high dose methotrexate (HD-MTX) based regimen, or a HD-MTX in combination with a high dose cytarabine. Addition of thiotepa and rituximab to MTX/cytarabine (MATRix regimen) increases the overall response (OR) and complete remission (CR) rates [12]. The progression free (PFS) and the overall survival (OS) rates also increase after consolidation with an autologous hematopoietic stem cell transplantation (Auto-HSCT) or WBRT in those treated with MATRix regimen compared to those receiving only MTX/cytarabine [13]. However, treatment with a thiotepa-based regimen in countries with a limited resource is difficult to implement. We, therefore, conducted a retrospective study of newly diagnosed PCNSL patients with a DLBCL subtype, who had received a HD-MTX-based chemotherapy, WBRT, or both for the treatment outcome and prognostic factors.

## Materials and methods

### Patients

Forty- three adult patients with a newly diagnosed PCNSL of DLBCL subtype at our hospital between May 2010 and May 2017 were analyzed. All histo-pathological and immuno-histochemical (IHC) slides were reviewed. The diagnosis of DLBCL was confirmed by an experienced hemato-pathologist, who categorized them by the Han’s algorithm [4, 14]. This subtype with MYC (≥40%) and Bcl2 (≥50%) co-expression was classified as double-expressor lymphoma (DEL) [15]. CD10, Bcl6 and MUM1 staining were considered positive when expressed in at least 30% of tumor cells. Characteristics of the brain lesion including number, area, sizes and leptomeningeal involvement were done by magnetic resonance imaging (MRI). Staging evaluation with computer tomogram of the chest and abdomen, and bone marrow biopsy were done in all patients. Excluded from analysis were those with the following features:- < 18 years old, positive serology for human immunodeficiency virus (HIV), secondary CNS lymphoma, post hematopoietic stem cell transplantation (HSCT) and treatment refusal.

### Study design and end points

#### Treatment protocol and evaluation

Fit patients aged ≤60 years old were treated with a HD-MTX (2–3 g/m^2^) on day 1 and cytarabine 2 g/m^2^ every 12 h on days 2 and 3, while those aged > 60–65 years received a reduced cytarabine dose at 1 g/m^2^. Those aged > 65 years or unfit received only HD-MTX at 1–2 g/m^2^on day 1 or WBRT. The chemotherapy was repeated every 2–3 weeks in the HD-MTX group, and every 3–4 weeks in the HD-MTX plus cytarabine one. Six and 3–4 cycles of chemotherapy were given to those receiving chemotherapy and a combined-modality therapy, respectively. Forty five and 36 Grey (Gy) of WBRT were given during 2010–2013 and 2014–2017, respectively. In those treated with combined-modality, they were given after completion of the planned chemotherapy. MRI of the brain was repeated after 3 cycles of chemotherapy and after completion of all treatment plans.

#### Primary end points

The objectives of the study were to analyze rates of relapse-free survival (RFS) and OS. A relapse-free survival was defined as the duration between date of CR and first relapse. An overall survival was defined as the duration between date of diagnosis and death.

#### Secondary end points

To evaluate the OR and CR rates, and identify factors which affect CR and survival. The overall response rate (ORR) was defined as percentage of patients with partial (reduction in tumor size > 50%) and complete remission in the whole group.

#### Statistical analysis

Age, gender, number size and area of brain lesion, serum LDH level and treatment regimen of patients with and without CR were compared using a Chi-square test. Kaplan-Meier and the log-rank test methods were performed for OS, RFS analysis and difference between groups (univariate analysis). Cox’s regression model was applied for a multivariate survival analysis. The SPSS statistics version 17 (Chicago: SPSS Inc.; 2008) was utilized for all data analysis. *P* < 0.05 was considered significant.

## Results

Patients’ characteristics and results of brain tissue pathology are shown in Tables 1 and 2, respectively. There were 43 patients with a median age of 65 years (range, 34–89 years), 24 of whom were male. Twenty three patients (53.5%) had an ECOG score > 2. Twenty seven (62.8%) and 31 patients (72%) had multiple brain lesions (≥ 2 lesions in one or more areas of brain involvement) and deep structure involvement, respectively. Ten patients (23.3%) had a maximum tumor diameter (MTD) ≥5 cm (median 3 cm and range 1–7.1 cm). A significantly larger tumor (≥5 cm) was observed in patients with solitary lesion (7/16 patients), compared to those with multiple ones (3/27 patients) (*p* = 0.014). MRI of the brain showed concurrent intra-ocular and leptomeningeal involvement in 1 and 2 patients, respectively. Serum LDH levels at diagnosis were recorded in 38 patients, 66% of whom had a normal value.

**Table 1:** Patients’ characteristics and treatment outcomes in 43 adult patients with PCNSL

Factor	ORR N (%)	*P*	CR N (%)	*P*	Median RFS (Mo)	*P*	Median OS (Mo)	*P*
Age (years)								
- < 60	10/12 (83)	0.966	4/12 (33)	0.065	NR	0.837	15	0.347
- ≥ 60	26/31 (84)		20/31 (65)		NR		17	
ECOG score								
- 0–2	18/20 (90)	0.298	15/20 (75)	0.018	NR	0.820	NR	0.001
- 3–4	18/23 (78)		9/23 (39)		NR		13	
Brain lesion								
- Single	13/16 (81)	0.735	5/16 (31)	0.013	NR	0.456	9	0.010
- Multiple (≥ 2)	23/27 (85)		19/27 (70)		NR		26	
Area of brain involvement								
- Single	12/15 (80)	0.629	4/15 (27)	0.005	NR	0.900	10	0.023
- Multiple (≥ 2)	24/28 (86)		20/28 (71)		NR		26	
Deep structure involvement								
- Yes	25/31 (81)	0.380	18/31 (58)	0.633	NR	0.141	26	0.203
- No	11/12 (92)		6/12 (50)		10		12	
Maximum tumor size								
- < 3 cm	14/17 (82)	0.844	10/17 (59)	0.748	NR	0.850	NR	0.103
- ≥ 3 cm	22/26 (85)		14/26 (54)		NR		13	
Maximum tumor size								
- < 5 cm	29/33 (88)	0.180	20/33 (61)	0.250	NR	0.217	26	0.004
- ≥ 5 cm	7/10 (70)		4/10 (40)		7		8	
Treatment								
- Chemotherapy (CMT)	12/14 (86)	0.028	5/14 (36)	0.056	NR	0.663	17	0.259
- Radiotherapy (RT)	5/9 (56)		4/9 (44)		NR		13	
- CMT + RT	19/20 (95)		15/20 (75)		NR		NR	
Treatment								
- CMT + RT	19/20 (95)	0.062	15/20 (75)	0.018	NR	0.845	NR	0.231
- Single therapy (CMT or RT)	17/23 (74)		9/23 (39)		NR		14	
LDH								
- Normal	22/25 (88)	0.681	16/25 (64)	0.544	NR	0.788	17	0.876
- High	12/13 (92)		7/13 (54)		NR		15	

*Abbreviations*: *ECOG* Eastern Co-operative Oncology Group, *LDH* lactate dehydrogenase, *ORR* overall response rate, *CR* complete remission rate, *PFS* progression free survival, *OS* overall survival, *NR* not reached

**Table 2:** The immuno-histochemical findings and treatment outcomes in 43 adult patients with PCNSL

Factor	ORRN (%)	*P*	CRN (%)	*P*	Median RFS (Mo)	*P*	Median OS (Mo)	*P*
Subtype of DLBCL								
- GCB	8/9 (89)	0.637	7/9 (78)	0.136	NR	0.985	NR	0.003
- Non GCB	28/34 (82)		17/34 (50)		NR		14	
BCL6								
- Positive	30/37 (81)	0.244	19/37 (51)	0.143	NR	0.842	17	0.534
- Negative	6/6 (100)		5/6 (83)		NR		12	
Non GCB								
- Positive MUM1	25/31 (81)	0.401	14/31 (45)	0.070	NR	0.323	14	0.290
- Negative MUM1	3/3 (100)		3/3 (100)		NR		NR	
BCL2								
- Positive	32/39 (82)	0.354	21/39 (54)	0.417	17	0.075	15	0.271
- Negative	4/4 (100)		3/4 (75)		NR		NR	
BCL2+ in GCB	5/6 (83)	0.710	4/6 (67)	0.368	NR	0.255	NR	0.061
BCL2+ in non GCB	27/33 (82)		17/33 (52)		NR		14	
MYC ≥40%								
- Yes	10/10 (100)	0.111	6/10 (60)	0.761	NR	0.628	17	0.896
- No	26/33 (79)		18/33 (55)		NR		15	
Double-expressor								
- Yes	9/9 (100)	0.137	5/9 (56)	0.986	NR	0.628	14	0.834
- No	27/34 (79)		19/34 (56)		NR		17	
Double-expressor								
- BCL6+	8/8 (100)	0.167	4/8 (50)	0.714	11	0.374	14	0.503
- BCL6-	28/35 (80)		20/35 (57)		NR		17	
Ki67 ≥ 80%								
- Yes	27/33 (82)	0.539	18/33 (55)	0.761	NR	0.790	17	0.897
- No	9/10 (90)		6/10 (60)		NR		14	

*Abbreviations*: *GCB* germinal center B cell, *ORR* overall response rate, *CR* complete remission rate, *PFS* progression free survival, *OS* overall survival, *NR* not reached

Positive staining for CD10, Bcl6, MUM1, Bcl2 and MYC were found in 19, 86, 91, 91 and 23%, respectively. GCB subtype was found in 9 patients (21%), 8 of whom were CD10+ (CD10+/Bcl6+/MUM1+) and 1 was Bcl6+/MUM1-. Eight GCB patients with CD10+ staining had two characteristic patterns of brain involvement; 6 had more than 1 area of involvement (2–3 areas) with the largest tumor diameter ≥ 2 cm, and the remaining 2 patients had solitary lesion in one area of involvement with the largest tumor diameter < 2 cm. One patients with Bcl6+/MUM1- had 4 areas of involvement with the largest tumor diameter < 1.5 cm. Deep structure involvement was found in 8 out of 9 GCB patients. In 34 patients (79%) with a non-GCB subtype, Bcl6+/MUM1+, Bcl6−/MUM1+ and Bcl6−/MUM1- were found in 65% (28/43), 7% (3/43) and 7% (3/43), respectively. Double-expressor lymphoma and double-expressor with positive Bcl6 staining were observed in 9 (21%) and 8 (19%) patients, respectively, and all of whom were found in non-GCB subtype. Thirty four patients (79%) had a triple protein expression of Bcl6, Bcl2 and MUM1. In non GCB patients, 21 (62%), 23 (68%), 24 (71%) and 9 (26%) patients had multiple area of involvement, deep structure involvement, the largest tumor diameter ≥ 3 and ≥ 5 cm, respectively.

**Fig. 1:**
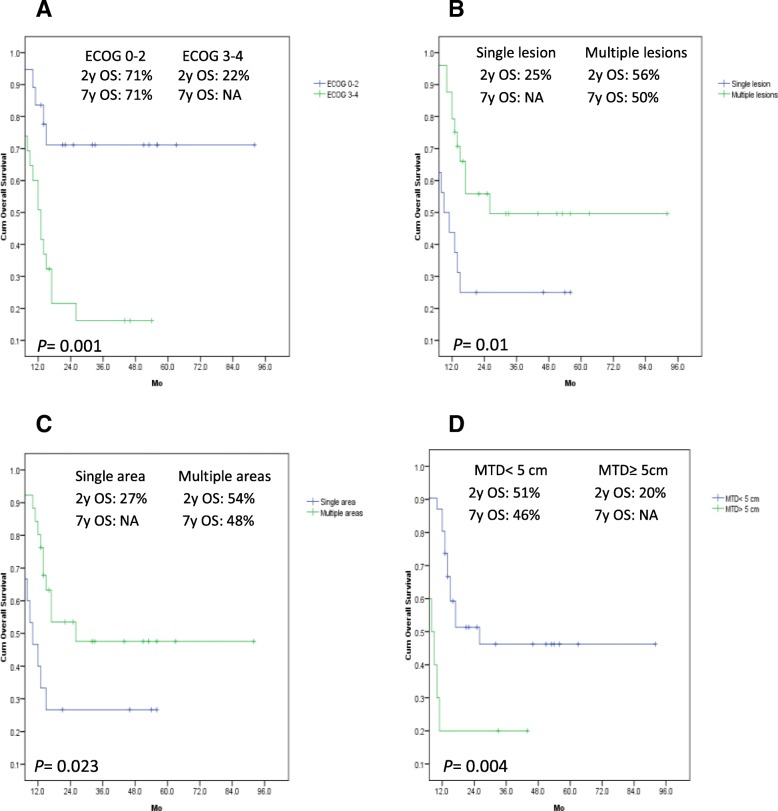
OS in 43 PCNSL patients with an ECOG score ≤ 2 and > 2 (**a**), with a solitary and multiple brain lesions. (≥2 lesions) (**b**), with a single and multiple area of brain involvement (**c**), with a MTD ≥5 and < 5 cm. (**d**)

**Fig. 2:**
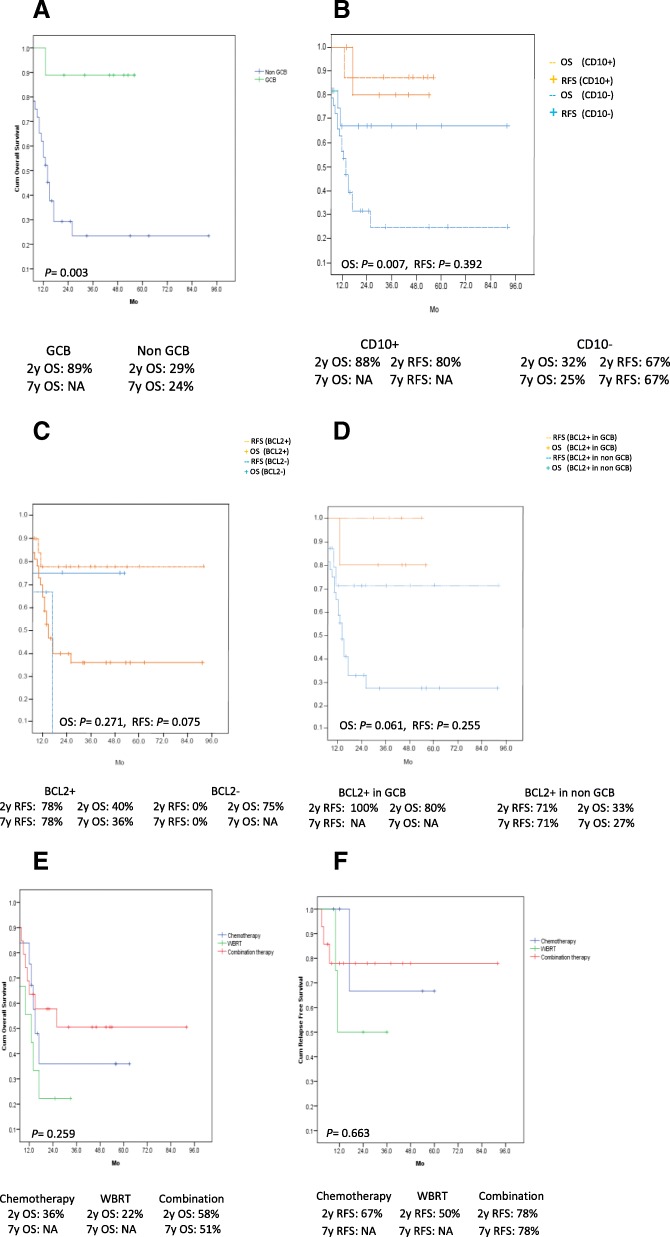
OS in 43 PCNSL patients with a germinal center B cell (GCB) and a non-GCB DLBCL (**a**), OS and RFS in 43 PCNSL patients with CD10+ and CD10- (**b**) OS and RFS in 43 PCNSL patients with BCL2+ and BCL2- (**c**), OS and RFS in 43 PCNSL patients with BCL2+ in GCB and BCL2+ in non GCB (**d**), OS in 43 PCNSL patients treated with chemotherapy, WBRT and chemotherapy combined with WBRT (**e**), RFS in 43 PCNSL patients treated with chemotherapy, WBRT and chemotherapy combined with WBRT (**f**)

A combined-modality therapy, chemotherapy and WBRT were given to 20 (46.5%), 14 (32.6%) and 9 (21%) patients, respectively. Patients’ characteristics of these 3 groups, incidentally, are not statistically different. Fourteen of the twenty patients (70%) treated with a combined-modality received a HD-MTX plus cytarabine, while the remaining 6 (30%) received only a HD-MTX. In the chemotherapy alone group, a HD-MTX and a HD-MTX/cytarabine were given in 8 (57%) and 6 (43%) patients, respectively. Only 3 patients received rituximab, 2 and 1 in the chemo- and the combined-modality therapy group, respectively. The ORR of the combined-modality therapy group was 95%, which was higher than those of the others, the chemotherapy being 86% (*p* = 0.383) and WBRT being 56% (*p* = 0.009). Overall CR rate was 57% and was better in patients receiving a combined-modality therapy (75%) when compared to those treated with only chemotherapy (36%, *p =* 0.036) or WBRT (44%, *p =* 0.109). In a univariate and multivariate analyses, a combined-modality therapy (*p* = 0.018), ECOG score > 2 (*p* = 0.018), number of brain lesion ≥2 (*p* = 0.013) and number of area of brain involvement ≥2 (*p* = 0.005) significantly affected the CR rate. The median follow-up time was 17 months, and the 7y-OS and RFS were 40 and 70%, respectively. Twenty four patients were dead, 18 of whom from a progressive disease, while the remaining 4 and 2 from infection and neurotoxicity, respectively. The median OS has not been reached in patients treated with the combined-modality, while it was 17 and 13 months (*p* = 0.259), respectively, in those treated with either chemotherapy or WBRT. The 2y-RFS in patients treated with the combined-modality, chemotherapy and WBRT were 78, 67 and 50%, respectively (*p* = 0.663). Factors associated with a prolonged OS in the univariate analysis were an ECOG score ≤ 2 (*p* = 0.001), a multiple brain lesions (*p* = 0.010), a multiple area of brain involvement (*p* = 0.023), a MTD < 5 cm (*p* = 0.004), a GCB subtype (*p* = 0.003), a positive CD10 staining (*p* = 0.007), and a Bcl2+ in GCB or Bcl2- DLBCL (*p* = 0.041). However, only an ECOG score ≤ 2 (*p* = 0.010) and a MTD < 5 cm (*p* = 0.011) were significantly associated with a better OS in the multivariate analysis. A Bcl6−/MUM1+ DLBCL and a Bcl2+/Bcl6−/MUM1+ DLBCL had a significantly shorter RFS. (Table 2) The survival curves are shown in Fig. 1 and 2. There was no statistically significant difference in survival between cases with myc less than or greater than 40% nor ki67 less than orgreater than 80%. Delayed neurotoxicity complication (14 and 18 months, respectivelyafter WBRT) was found in 2 out of 20 (10%) in the combined-modality group, with one each receiving 45 Gy and 36 Gy of WBRT, respectively.

## Discussion

Our study showed that both chemotherapy and chemotherapy combined with WBRT induced a high response rate (ORR = 86 and 95%, respectively). However, the improved CR rate (75%) was found only in those treated with a combined-modality one, similar to that reported by the International Extra-nodal Lymphoma Study Group-32 (IELSG-32), which showed a low CR rate after treating with 4 cycles of either HDMTX/Cytarabine (23%) or MATRix (49%) regimen. However, adding a 36 Gy of WBRT after MATRix increased the CR rate to 95% [13]. A non-statistically different (*p =* 0.109), CR rate between those receiving a combined-modality therapy (75%) and WBRT (44%) in our study was later eclipsed by the high relapse rate in the latter group (Fig. 2). Although our patients did not receive a rituximab- or a thiotepa-based regimen, the treatment outcome was not different from those reported earlier [9–12]. The RFS and OS curves of our patients reached plateau after 2 years of initial treatment. Rate of RFS (70%) was better than that of OS (40%), because many patients, who didn’t achieve a CR, had disease progression and died. Those achieving and sustaining a CR status within 2 years after treatment had a prolonged disease-free survival. Both RFS and OS rates were highest in those receiving a combined-modality therapy. However, this was mitigated by its 10% late neurotoxicity, which was unacceptable. Since one of the two cases had already received a lower radiation dose, together with no neurotoxicity in both single-modality therapy groups, the result suggested that modification of both modalities is needed to reduce this tragic complication. However, it must be done with a minimal or no sacrifice to the treatment efficacy. For the time being, this preliminary results suggested that a combined-modality therapy may be the best treatment option for PCNSL in countries with a limited access to the thiotepa-based regimen.

Multiple brain lesions were found in 63% of our patients, similar to those previously reported [13, 16]. However, only 20–40% prevalence were also reported [17–21]. Large tumor (≥5 cm diameter) was found, particularly in patients with a solitary brain lesion, and had a worse CR and OS. Although the IELSG scoring system was not applicable due to lack of data on the CSF protein concentration, our study showed that an ECOG score ≤ 2, a multiple brain lesions, a MTD < 5 cm, a GCB subtype, a CD10+ were significantly associated with a prolonged OS. Interestingly, Han et al. [4] also reported that expression of CD10 protein was associated with a better OS in systemic DLBCL. Although MUM1 protein was co-expressed with CD10+ in the brain tissue of our 8 GCB patients (CD10+/Bcl6+/MUM1+), the survival benefit was still seen, which has never been observed previously. Currently, the outcome of CD10+/MUM1+ in systemic DLBCL patients are not yet known [4, 22–24]. Furthermore, the Bcl6+ non-GCB tends to have a better RFS and OS than the Bcl6- counterpart. Triple negative CD10/BCL6/MUM1 (non GCB) was seen in 3 patients and, interestingly, they had a better RFS and OS than the MUM1+ one. Expression of Bcl2 protein was associated with a significantly worse OS in both the Bcl6+ and Bcl6- non-GCB group. The GCB subtype exhibited an excellent OS and RFS, even when co-expressed with Bcl2 protein. The median follow-up time of our patients was short, because many, who failed to achieve a CR, died early. Limitations of our study were a retrospective one, and a small number of patients.

### Conclusions

PCNSL had a dismal OS. Factors associated with a prolonged survival in this study were cell of origin of DLBCL (GCB subtype), lesion characteristics (MTD < 5 cm, multiple brain lesions, multiple area of brain involvement), patients’ performance status (ECOG score ≤ 2) and treatment regimen (chemotherapy combined with WBRT).

## Data Availability

Not applicable.

## Abbreviations

DEL: Double-expressor lymphoma
DLBCL: Diffuse large B cell lymphoma
ECOG: Eastern co-operative oncology group
GCB: Germinal center B cell
MTD: Maximum tumor diameter
PCNSL: Primary central nervous system lymphoma
